# Diversity and abundance of antibiotic resistance genes and their relationship with nutrients and land use of the inflow rivers of Taihu Lake

**DOI:** 10.3389/fmicb.2022.1009297

**Published:** 2022-10-04

**Authors:** Prilli Arista Fernanda, Shuang Liu, Tianma Yuan, Bharathi Ramalingam, Jing Lu, Raju Sekar

**Affiliations:** ^1^Department of Biological Sciences, Xi’an Jiaotong-Liverpool University, Suzhou, China; ^2^Suzhou Xishan Biotechnology Inc. (VRL Asia), Suzhou, China; ^3^Marie Skłodowska-Curie Actions, SDGine for Healthy People and Cities, Department of Forestry and Environmental Management, Technical University of Madrid (UPM), Madrid, Spain

**Keywords:** antibiotic resistance genes (ARGs), high-throughput profiling, water quality, land use, inflow rivers, Taihu Lake

## Abstract

Taihu Lake is the third largest freshwater lake in China and an important source for drinking water, flood protection, aquaculture, agriculture, and other activities. This lake is connected to many principal and small rivers with inflow from west and outflow on the eastern side of the lake and these inflow rivers are believed to significantly contribute to the water pollution of the lake. This study was aimed at assessing the diversity and abundance of antibiotic resistance genes (ARGs) and mobile genetic elements (MGEs), and their relationship with water quality parameters and land use patterns. Water samples were collected from 10 major inflow rivers and the source water protection area of the Taihu Lake in spring and summer 2019. High-throughput profiling was used to detect and quantify 384 ARGs and MGEs and in addition, 11 water quality parameters were analyzed. The results showed that the number of ARGs/MGEs detected in each inflow river ranged from 105 to 185 in spring and 107 to 180 in summer. The aminoglycoside resistance genes were the most dominant types ARGs detected followed by beta-lactam resistance, multidrug resistance, macrolide-lincosamide-streptogramin B (MLSB) resistance genes, which contributed to 65% of the ARGs. The water quality parameters showed significant correlation with absolute abundance of ARGs. Furthermore, significant correlation between ARGs and MGEs were also observed which demonstrates potential gene transfer among organisms through horizontal gene transfer *via* MGEs. ARGs showed strong positive correlation with cultivated and industrial lands whereas, negative correlation was observed with river, lake, forest, land for green buffer, and land for port and harbor. The overall results indicate that the inflow rivers of Taihu Lake are polluted by various sources including multiple nutrients and high abundance of ARGs, which needs attention for better management of the inflow rivers of this lake.

## Introduction

Antibiotics are widely used in many sectors including health care, agriculture, and livestock industries (Martinez, 2009; Fair and Tor, 2014; Van Den Honert et al., 2018). The overuse or abuse of antibiotics in various industries accelerates the bacterial ability to adapt to high concentrations of antibiotics and to develop resistance against single or multiple antibiotics, which makes the treatment ineffective (Van Den Honert et al., 2018). World Health Organization classified antibiotic resistance as one of the most threats to global public health due to the quick spread of antibiotic resistance, which can be seen through the detection of antibiotics, antibiotic resistant bacteria (ARB), and antibiotic resistance genes (ARGs) in a wide range of environments such as soil, sediments, surface water, wastewater, and drinking water (Bergeron et al., 2015; Liu et al., 2018; Huang et al., 2019; Voigt et al., 2020; Seyoum et al., 2021). The aquatic environments are considered as an ideal medium for proliferation of ARB and spread of ARGs (Young, 1993; Suzuki et al., 2017; Na et al., 2018). High abundance of ARB and ARGs have been detected in the aquatic environments, particularly in rivers and lakes (Amos et al., 2014; Zheng et al., 2017; Zheng et al., 2018; Chen et al., 2019; Nnadozie and Odume, 2019; Liang et al., 2020). As bacteria exposure at extremely low antibiotic concentrations is sufficient to maintain the resistance, human and animals are prone to be exposed to ARB and ARGs by direct handling and consumption of contaminated water and aquaculture products carrying resistance genes (Tao et al., 2010; Gullberg et al., 2011; Larsson et al., 2018; Rousham et al., 2018). Thus, the ARB and ARGs are eventually transmitted from environment to human and animals and vice versa. Various factors including the water temperature, flow dynamics and the concentrations of nutrients, heavy metals, antibiotics, and the land use were reported to contribute to the diversity and abundance of ARGs in water environment (Walsh et al., 2011; Yang Y. et al., 2017; Ohore et al., 2019; Zhang M.-S. et al., 2021; Yang et al., 2022).

A recent survey showed that the worldwide antibiotic consumption was increased by 60% from 2005 to 2015, which was mainly caused by increased antibiotic consumption by the low-middle income countries such as India, China, Pakistan, Tunisia, and Indonesia (Klein et al., 2018). China is one of the largest producers and consumers of antibiotics in the world and previous reports showed that the yearly consumptions of antibiotics were in the range of 162–180 kilotons and they were mainly used in human medicine, livestock, and agriculture (Luo et al., 2010; Zhang Q.-Q. et al., 2015). Nearly 80–90% of the consumed antibiotics finally end up in the environment through various wastes containing human and animal feces and urine and they contribute to the increased levels of antibiotic resistant pathogens in the environment. In China, the rural areas in particular were reported to contribute more to this problem due to limited infrastructure to treat sewage (GOSC, 2012) and lack of specific standard to guide discharge of the livestock wastes to the environment therefore, the discharge of livestock wastes into rivers and lakes and the application of wastes to agricultural land became a common practice (Zhou et al., 2011). The presence of high concentration of antibiotics in water bodies has led to serious public health problems due to microbe’s ability to uptake and promotes resistance to antibiotics. In recent years, a number of studies investigated the ARB and/or ARGs in water environment in China ranging from tap water to surface water and sediments in lakes and rivers (Xu et al., 2016; Yan et al., 2017; Zhou et al., 2017; Liu et al., 2018; Zheng et al., 2018; Chen et al., 2020; Jiang et al., 2021). Particularly, the quantification of ARGs in freshwater environments received more attention recently due to its importance for drinking, transportation, agricultural, and other activities (Xiong et al., 2015; Zheng et al., 2017; Huang et al., 2019; Jiang et al., 2021).

Taihu Lake is the third largest freshwater lake in China, located in southeast Jiangsu Province. This lake is located in rapid industrial development regions with high rate of anthropogenic activities combined with agricultural areas and economic development (Qin et al., 2007; Stange et al., 2019). More than 10 principal rivers and 200 small rivers are connected to the Taihu Lake with water inflows from west side of the lake and outflow in the eastern side of the lake (Qin et al., 2007; Vadde et al., 2018). Taihu Lake is important as water resource for drinking water for several large cities, flood protection, aquaculture, agriculture, tourism, and transportation. With the fact that high dense population with high anthropogenic activities surrounds Taihu Lake, water pollution such as excessive algal growth and microbial contaminants cannot be avoided. Previous studies have reported that influx of high nutrients from inflow rivers affected the water quality of Taihu Lake severely, especially from Taige Canal, Chendong River, and Tiaoxi River (Qin et al., 2007; Du et al., 2017; Vadde et al., 2018; Jiang et al., 2021). Besides that, dense population surrounding Taihu Lake also contributes to the dissemination of antibiotic resistance through overuse of antibiotic in the hospital, animal livestock, and agricultural field. Many studies have reported the detection of ARGs in Taihu Lake (Han et al., 2013; Yin et al., 2013; Zhang S. H. et al., 2015; Stange et al., 2019) and have evaluated the water quality of some rivers connected to Taihu Lake (Dai et al., 2013; Du et al., 2017). However, the studies focusing on the detection of ARB and ARGs in the inflow rivers are scarce (Zheng et al., 2017; Jiang et al., 2021). Zheng et al. (2017) studied the abundance of ARGs in East Tiaoxi River and Jiang et al. (2021) focused on the detection of ARGs and mobile genetic elements (MGEs) in Taige canal. It has been reported that the systematic monitoring of ARGs in rivers in China is still rare and the information on the spatial distribution and seasonal variation of ARGs in rivers and their relationship to the environmental factors is lacking (Jiang et al., 2021). Moreover, it appears that most of the previous studies on ARGs focused on the urban rivers and less emphasis were given to the rural rivers which are affected by the agriculture pollution (Huang et al., 2019; Jiang et al., 2021). Therefore, this study was aimed at investigating the diversity and abundance of ARGs in 10 principal inflow rivers (which flow through rural and/or urban areas) by high-throughput profiling and the spatial distribution/seasonal variations in ARGs and their relationship with water quality/land use pattern. The findings from this study reveals the water quality and the level of ARGs in the inflow rivers of Taihu Lake for better management of the rivers and the lake.

## Materials and methods

### Sampling sites and sample collection

**FIGURE 1:**
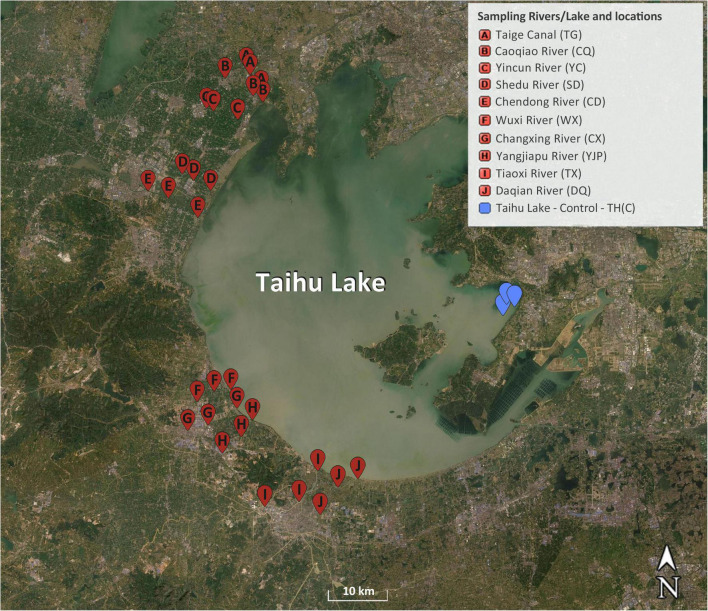
Map of sampling locations in the inflow rivers and the protected area (control locations) of Taihu Lake. Adapted from Earth Online (n.d.).

Ten inflow rivers in the northwest and southwest side of Taihu Lake were chosen for this study and the water samples were collected from three locations in each inflow river (Figure 1). The samples were collected from following inflow rivers, which flow in to the Taihu Lake from west side of the lake: Taige Canal, Caoqiao River, Yincun River, Shedu River, Chendong River, Yangjiapu River, Wuxi River, Changxing River, Tiaoxi River, and Daqian River. Previous reports showed that some of these rivers contributed highly to the water quality and pollution of the Taihu Lake, due to dense population, farming, and industrial activities in the surrounding area (Qin et al., 2007; Vadde et al., 2018). Three sampling locations in the source water protection area of the Taihu Lake in the east side of the lake were selected as control locations for this study. The samples were collected in two seasons (spring and summer 2019) and 66 samples in total. Ten liters of surface water samples were collected from each location using sterile high density polyethylene (HDPE) containers and the samples for nutrients and microbiological analyses were kept on ice until the samples were brought to the laboratory. The samples were processed within 24 h of samples collection.

### DNA extraction

Five hundred milliliter of water samples were filtered by vacuum filtration using sterile 0.22 μm pore size polycarbonate membrane filters (Millipore, St. Louis, MO, USA) and stored at −80°C until further processing. The membrane filter for each sample was sliced into small pieces and the DNA was extracted using DNeasy PowerSoil kit (Qiagen, Hilden, Germany). The quality and quantity of the DNA was measured using Nanodrop spectrophotometer and the extracts were stored at −80°C until further processing. Triplicate DNA extracts with good quality and quantity within each inflow river were pooled together for further molecular analyses.

### Quantification of antibiotic resistance genes by high-throughput qPCR

High-throughput qPCR (HT-qPCR) was performed in order to profile the presence and abundance of resistance genes in DNA extracted from the water samples and the mechanism of their action. The high-throughput (HT)-ARGs profiling was carried out at the Institute of Urban Environment, Chinese Academy of Sciences, Xiamen, China. A total number of 384 ARGs/MGEs along with 16S rRNA genes were targeted in this study (Stedtfeld et al., 2018; Supplementary Table 1). The qPCR assays and the data analyses were carried as per the method described previously (Xu et al., 2016). The HT-qPCR amplification of the 16S rRNA genes was conducted with a total volume of 20 μL, consist of 10 μL 2 × LightCycler 480 SYBR^®^ Green I Master Mix (Roche Inc., Wilmington, MA, USA), 5.9 μL of nuclease-free water, 0.5 μL of 50 mg/mL BSA, 2 μL of 40 ng/μL DNA templates, and 0.8 μL of 10 μM of each forward and reverse primer. The amplification was conducted with Roche 480 (Roche Inc., Wilmington, MA, USA) with pre-incubation at 95°C for 5 min, followed by 40 cycles of 95°C for 15 s, annealing at 60°C for 1 min, and 72°C for 20 s. The amplification of ARGs was carried out with a total reaction volume of 100 nL reaction containing 1 × LightCycler 480 SYBR^®^ Green I Master Mix (Roche Inc., Wilmington, MA, USA), nuclease-free PCR-grade water, 1 ng/μL BSA, 4 ng/μL DNA templates, and 1 μM of each forward and reverse primer. The thermal cycle consists of initial denaturation at 95°C for 10 min, followed by 40 cycles of denaturation at 95°C for 30 s, annealing at 60°C for 30 s and lastly a melting curve analysis was auto generated by the program. The 16S rRNA and ARGs copies were determined according to Ct value and based on the original DNA concentration used for qPCR, elution volume and amount of water samples filtered for the ARGs quantification. The number of relative and absolute abundance of ARGs from each sample was calculated.

### Physico-chemical analysis of water samples

The air temperature, water temperature and conductivity were measured on-site using a portable EC/TDS TEMP Waterproof Combo Meter (C-100, HM Digital Inc., Culver City, CA, USA). The pH was measured using a portable Eutech pH700 meter (Thermo Fisher Scientific Inc., Waltham, MA, USA). One hundred ml of water sample in triplicate was filtered using glass fiber filters (Millipore, St. Louis, MO, USA); Chlorophyll *a* (Chl *a*) was extracted using 90% acetone and measured by spectrophotometry following the method of American Public Health Association (APHA, 2005). The samples for nutrient analysis were stored at −20°C soon after arrival to the lab and the parameters such as total nitrogen (TN), total phosphorus (TP), nitrate nitrogen (NO_3_-N), nitrite nitrogen (NO_2_-N), ammonium nitrogen (NH_4_-N), phosphate (PO_4_-P), and total organic carbon (TOC) were measured as reported in our earlier studies (Vadde et al., 2018; Yuan et al., 2019). All the values of physico-chemical parameters were compared to the class III water quality standards set by the Chinese Ministry of Environmental Protection (now called as Ministry of Ecology and Environment, People’s Republic of China) (MEP, 2002) and correlated with the results of ARGs.

### Land-use pattern analysis

The land-use maps were prepared using the geographical information system (GIS) software ArcGIS 10.3 and ArcGIS Pro. Based on the Google Earth China Service Map of cities around Taihu Lake, a layer of buffer zones with a radius of 1,000 m around each sample point was created in April 2021. By referencing the official existing and proposed land use maps of Changzhou, Wuxi, and Huzhou cities where sampling points were located, as well as Google Earth and Baidu maps covering the sample areas, the detailed land-use types, were digitized within these buffer zones in accordance with the national *Code for classification of urban land use and planning standards of development land* (GB50137-200) (MOHURD, 2011) and the newly released national *Guidance of Territorial Spatial Investigation, Plan, Land use control Classification* (MOHURD, 2020). The classification percentages were averaged for three locations in each river to represent the land-use status and this was used in the correlation analyses.

### Statistical analysis

Basic statistical analysis was carried out on Microsoft Excel 2010 and Graphpad Prism 6. Variations in the physico-chemical parameters within the sampling locations and seasons were analyzed by two-way analysis of variance (ANOVA) at *p* < 0.05 level of significance. The correlations between ARGs/MGEs absolute abundance and (i) water quality parameters and (ii) land-use type percentages were studied by Spearman’s correlation analysis. The principal component analysis (PCA) showing the distribution of ARGs and MGEs were done using R (version 4.1.2) and R studio (version 1.3.1093). The correlation between the absolute abundance of ARGs and MGEs were carried by Pearson’s correlation analysis.

## Results

### Diversity and abundance of antibiotic resistance genes and resistant mechanisms

**FIGURE 2:**
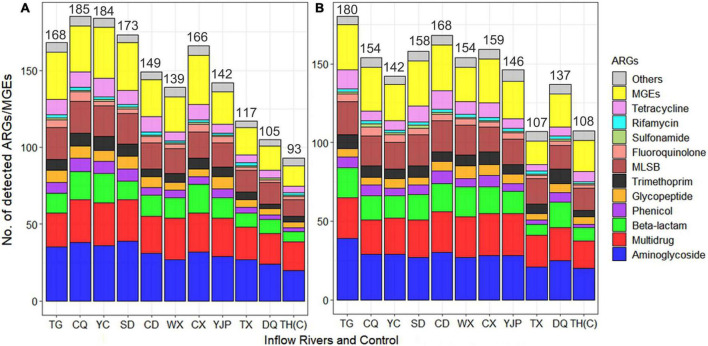
Average number of antibiotic resistance genes (ARGs) and mobile genetic elements (MGEs) detected in 10 inflow rivers and the protected area (control) of Taihu Lake during spring **(A)** and summer **(B)**. The resistance genes detected in all water samples were classified based on the antibiotic class to which they confer resistance.

A total of 384 ARGs/MGEs and 16S rRNA genes were tested using the DNA extracted from water samples. The number of ARGs detected in each inflow river ranged from 105 to 185 in spring and 107 to 180 in summer (Figure 2 and Supplementary Tables 2, 3). The inflow rivers Taige Canal (TG), Caqiao River (CQ), Yincun River (YC), Shedu River (SD), and Chendong River (CD) are located in the North-West (NW) region of the Taihu Lake whereas, Wuxi River (WX), Changxing River (CX), Yangjiapu River (YJP), Tiaoxi River (TX), and Daqian River (DQ) are located in the South-West (SW). In general, the NW region of rivers had much higher number of ARGs in both spring (172 ± 15) and summer (160 ± 14) as compared to rivers located in SW region (134 ± 24 and 141 ± 20 in spring and summer, respectively). In NW region of the rivers, in spring, significantly higher aminoglycoside (*p* < 0.01), phenicol (*p* < 0.05), glycopeptide (*p* < 0.05), MLSB (*p* < 0.01), and tetracycline (*p* < 0.05) resistance genes were observed as compared to SW region. However, in summer, only MLSB and fluoroquinolone resistance genes were significantly higher (*p* < 0.01) in NW region as compared to SW region. With respect to seasons, the number of ARGs detected in CQ, YC, SD, CX, and TX Rivers was higher in spring than summer. However, the rivers TG, CD, WX, YJP, DQ, and the control location TH(C) had higher ARGs in summer than spring.

**FIGURE 3:**
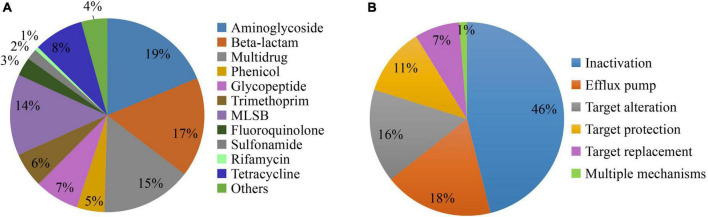
The diversity of antibiotic resistance genes (ARGs) detected in the inflow rivers classified based on the antibiotic class to which they confer resistance **(A)** and the observed mechanisms of resistance **(B)**.

The ARGs conferring resistance to aminoglycoside was the most dominant types of ARGs detected, followed by beta-lactam, multidrug, and macrolide-lincosamide-streptogramin B (MLSB) resistance genes which contributed to 65% of the ARGs detected (Figure 3A). The tetracycline, glycopeptide, trimethoprim, and phenicol resistance genes contributed to 26% of the ARGs. The ARGs conferring resistance to all antibiotic classes were detected in all inflow rivers in spring, except rifamycin in DQ and control locations. Among the 308 ARGs, five known resistance mechanisms were detected and among them antibiotic inactivation (46%) was the dominant mechanism followed by efflux pump (18%), target alteration (16%), target protection (11%), and target replacement (7%). Interestingly, four ARGs (*lmrR, tetR, acrR*, and *marR*) conferring resistance to MLSB, tetracycline, and multidrug, respectively, were found to have multiple mechanisms of resistance, i.e., by target alteration and efflux pump (Figure 3B).

**FIGURE 4:**
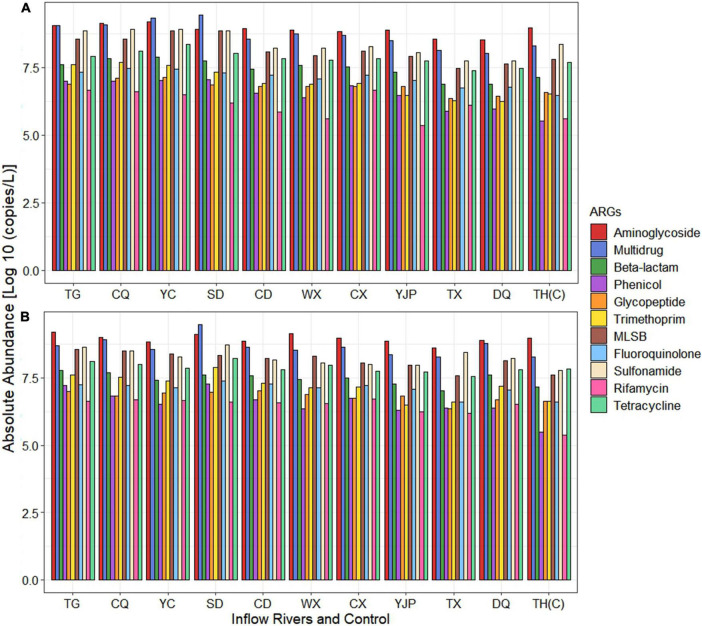
Absolute abundance of antibiotic resistance genes (ARGs) (grouped based on the antibiotic class to which they confer resistance) in inflow rivers and control location in **(A)** spring and **(B)** summer.

The absolute abundance of ARGs conferring resistance to different antibiotic classes is shown in Figure 4. The difference in the ARGs conferring resistance to most of the antibiotic classes did not show significant difference (*p* > 0.05) between spring and summer except rifamycin which showed significant difference (*p* < 0.01) between seasons (Supplementary Table 4). However, ARGs conferring resistance to five antibiotic classes (aminoglycoside, beta-lactam, phenicol, fluoroquinolone, and rifamycin) and multidrug showed statistically significant difference (*p* < 0.05) between locations (Supplementary Table 4). Various genes encoding for MGEs including transposase, insertional sequences, plasmid, and integrase were quantified in each inflow rivers (Supplementary Figure 1). The absolute abundance of ARGs conferring resistance to each antibiotic class was ranged between 0 and 9.44 Log10 copies/liter in spring and 6.17–9.47 Log10 copies/L, with multidrug as the highest (9.44 Log10 copies/L in spring and 9.47 Log10 copies/L in summer), followed by aminoglycoside (9.18 Log10 copies/L in spring and summer). The lowest abundance of ARGs was observed in rifamycin and phenicol. Furthermore, in spring, the absolute abundance of ARGs conferring resistance to ten antibiotic classes (except rifamycin) showed significantly higher abundance (*p* < 0.05) in rivers located in the NW region (TG, CQ, YC, SD, and CD rivers) as compared to SW region (WX, CX, YJP, TX, and DQ rivers) of the Taihu Lake. In summer, the significantly higher ARGs in NW region was observed only for six antibiotic classes (phenicol, glycopeptide, trimethoprim, MLSB, sulfonamide, and tetracycline).

The relative abundance of ARGs and MGEs in the inflow rivers and control location are shown in Supplementary Figures 2, 3. The evaluation of relative abundance of ARGs was done through normalizing all ARGs with the 16S rRNA gene abundance to ensure variations in DNA quantities were not affected with the variations in ARGs abundance. Generally, the average relative abundance of ARGs ranged between 2.0 × 10^–4^–6.063 × 10^–1^ copies/bacterial cell in spring and 3.0 × 10^–4^–9.184 × 10^–1^ copies/bacterial cell in summer, indicating each bacterial cells carrying 2.0 × 10^–4^–6.063 × 10^–1^ resistance genes in spring and higher resistance genes ranging 3.0 × 10^–4^–9.184 × 10^–1^ were present in single bacteria during summer. Out of all relative abundance of ARGs detected in water samples, only genes conferring resistance to rifamycin and sulfonamide showed significant seasonal (*p* < 0.05) variations whereas, genes conferring resistance to eight antibiotic classes showed significant (*p* < 0.05) spatial variations (Supplementary Table 4). Highest normalized gene copy number of tetracycline was observed in YC (0.0131 copies/cell), followed by SD River (0.007 copies/cell) and CQ river (0.0086 copies/cell). However, the relative abundance of tetracycline has declined in summer compared to spring, specifically on YC where relative abundance decreased to 0.0061 copies/bacterial cell. Yet, no significant (*p* > 0.05) seasonal variations were observed.

### Spatial distribution of antibiotic resistance genes and mobile genetic elements in spring and summer

**FIGURE 5:**
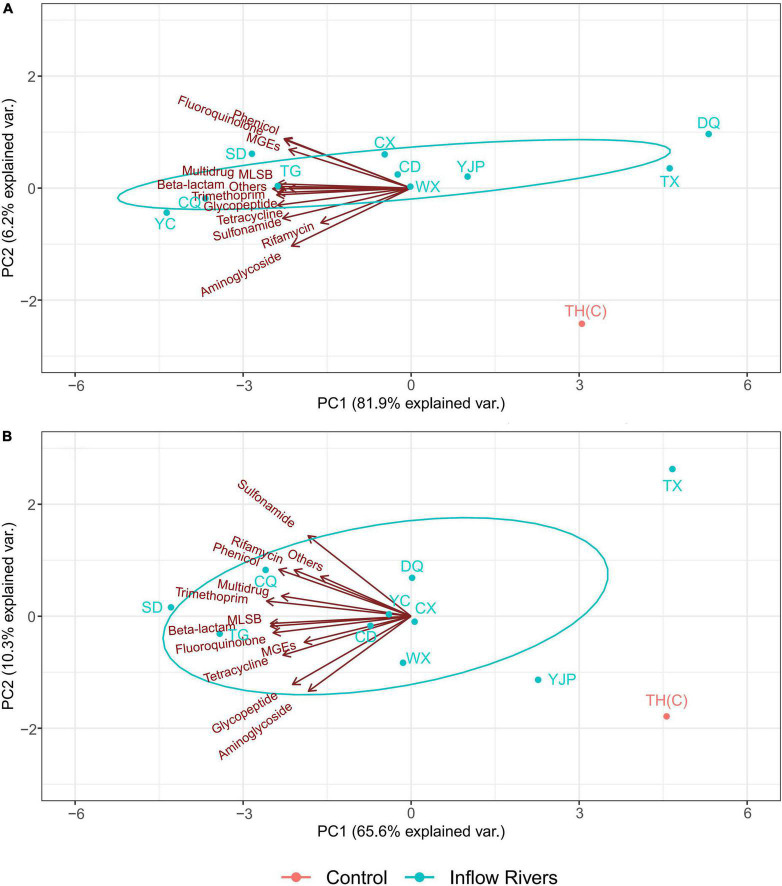
The spatial distribution of antibiotic resistance genes (ARGs) and mobile genetic elements (MGEs) in the inflow rivers and protected area of Taihu Lake in spring **(A)** and summer **(B)** 2019.

The spatial distribution of the ARGs and the MGEs in the inflow rivers and the control locations in spring and summer are shown in Figure 5. In spring, PC1 and PC2 explained 81.9 and 6.2% of the total variation, respectively. All the ARGs and MGEs affected the total variation mainly as negative loadings on PC1. The ARGs conferring resistance to phenicol and fluoroquinolone and MGEs contributed to positive loading on PC2 while aminoglycoside, rifamycin, and sulfonamide were the major contributors of negative loading on PC2. The ARGs and MGEs were most abundant in the inflow rivers TG, CQ, YC, and SD, moderately abundant in CX, CD, YJP, WX, and least abundant in TX, DQ, and TH (C) (Figure 5A).

In summer, PC1 and PC2 explained 65.6 and 10.3% of the total variations, respectively. PC1 had negative loadings of all the ARGs and the MGEs. Sulfonamide, phenicol, rifamycin, trimethoprim, multidrug resistance genes, and others contributed to positive loadings on PC2 while, aminoglycoside, glycopeptide, tetracycline, fuoroquinolone, beta-lactam, and MLSB resistance genes and MGEs had a negative loading on PC2. The ARGs and MGEs were most abundant in the inflow rivers TG and CQ and CD, moderately abundant in YC, WX, DQ, and CX rivers and least abundant in YJP, TX, and TH(C) (Figure 5B).

### Physico-chemical parameters at different locations and seasons

**FIGURE 6:**
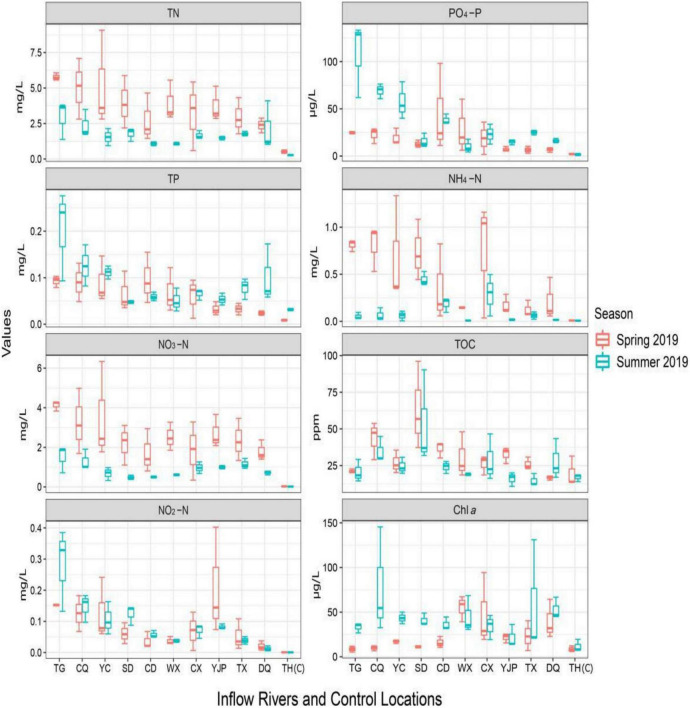
Seasonal and spatial variations in total nitrogen (TN), total phosphorus (TP), nitrate-N (NO_3_-N), nitrite-N (NO_2_-N), phosphate-P (PO_4_-P), ammonia-N (NH_4_-N), total organic carbon (TOC), and Chlorophyll *a* (Chl *a*).

The results of physico-chemical analysis of 10 inflow rivers and control locations of Taihu Lake measured in two seasons are shown in Figure 6 and Table 1 (range values, acceptable limit, and statistical results). The water temperature was significantly higher in summer as compared to spring. The conductivity values were high and varied between the sampling locations in spring (260–674 μS/cm) as compared to the values observed in summer (110–493 μS/cm). The pH values ranged from 7.35 to 7.78 in spring and 7.20–8.25 in summer and these values were within the acceptable range (6.5–8.5) set by the Ministry of Environmental Protection (MEP), China. Among the nutrient parameters tested, except TP, NO_3_-N, and NH_4_-N levels, all other parameters were outside the acceptable limit set by the MEP, China. The nutrient levels were generally higher in the rivers located in the NW region of the rivers as compared to the rivers located in the SW (Figure 6). TN values in all rivers sampled were very high as compared to the control locations and the values exceeded the acceptable limit (<1 mg/L). The TN values significantly varied between the rivers (spatial) and seasons with significantly higher values in spring (Table 1). The TP values also showed significant spatial and seasonal variations, however, the values were significantly lower in spring as compared to summer. The NO_2_-N levels were varied significantly between locations, however, the difference was insignificant between seasons although elevated levels were observed in spring as compared to summer. Both NO_3_-N and NH_4_-N levels significantly varied between the rivers sampled and the seasons, however, the values were within the acceptable limit (Table 1). Both PO_4_-P and Chl *a* levels were significantly high in summer than spring. No specific standards were set by MEP for both PO_4_-P and Chl *a*, however, PO_4_-P concentration below 20 μg/L was commonly detected in streams and river, and concentration value above 20 μg/L indicate water pollution (Shock et al., 2003). Similarly, no specific standard was set by MEP, China for TOC and an elevated TOC levels were observed in spring, the levels only showed statistically significant spatial difference (*p* < 0.0001).

**TABLE 1:** Summary of physico-chemical characteristics of water samples collected from ten inflow rivers and control locations in spring and summer 2019.

Parameters	Acceptable range (by MEP)	Control location (Spring–Summer)	Range (Min–Max)		*p*-value	
			Spring 2019	Summer 2019	Seasonal	Spatial
WT (°C)	–	26.8–32.1	16.4–22.7	30.0–32.8	<0.0001****	<0.0001****
pH	6.5–8.5	8.28–8.62	7.35–7.78	7.20–8.25	0.0099**	<0.0001****
Conductivity (μS/cm)	–	347–385	260–674	110–493	<0.0001****	<0.0001****
TN (mg/L)	1 mg/L	0.25–0.64	2.38–5.79	1.07–2.93	<0.0001****	0.0022**
TP (mg/L)	1 mg/L	0.01–0.03	0.02–0.10	0.05–0.20	0.0116*	<0.0001****
NO_2_-N (mg/L)	0.15 mg/L	0.0003–0.0007	0.02–0.21	0.01–0.28	0.6593	<0.0001****
NO_3_-N (mg/L)	10 mg/L	0.01–0.03	1.72–4.13	0.48–1.50	<0.0001****	0.0005***
NH_4_-N (mg/L)	1 mg/L	0.007–0.012	0.12–0.81	0.01–0.45	<0.0001****	0.0001***
PO_4_-P (μg/L)	–	0.89–2.89	6.59–44.55	10.08–108.11	0.0001***	<0.0001****
TOC (mg/L)	–	13.65–18.63	16.74–63.40	14.71–53.05	0.0651	<0.0001****
Chl *a* (μg/L)	–	8.68–11.70	8.59–55.15	21.72–77.56	0.0028**	0.0804

*Statistically significant difference at *p* < 0.05; **Statistically significant difference at *p* < 0.01; ***Statistically significant difference at *p* < 0.001; ****Statistically significant difference at *p* < 0.0001.

### Correlation between antibiotic resistance genes/mobile genetic elements and water quality parameters

**FIGURE 7:**
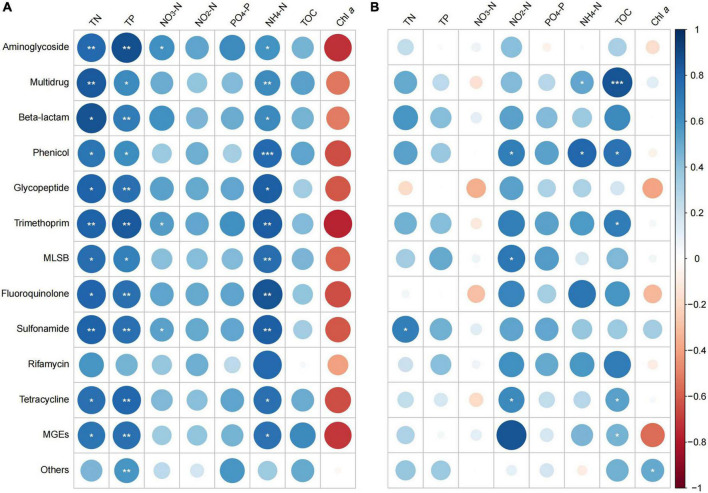
Spearman correlation between antibiotic resistance genes (ARGs)/mobile genetic elements (MGEs) and water quality parameters in **(A)** spring and **(B)** summer of 2019. Sizes of the bubbles indicate correlation coefficient (Larger bubbles means stronger correlations). Significance indicator: *p < 0.05; **p < 0.01; ***p < 0.001.

In general, stronger and more significant correlations were observed between ARGs and MGEs in spring, compared to that in summer. In spring, all the ARGs and MGEs showed positive correlations with TN, TP, NO_3_-N, NO_2_-N, PO_4_-P, NH_4_-N, and TOC, but negative correlations with Chl *a* levels (Figure 7A). Among all the correlations, the ARGs/MGEs correlated strongly and significantly with TN, TP, and NH_4_-N.

In summer, the correlation pattern was less obvious than that of in spring. There were no clear correlation patterns between ARGs/MGEs and NO_3_-N or Chl *a.* Both positive and negative correlations were found in other parameters. Positive correlations were found between most of the ARGs/MGEs and TN, TP, NO_2_-N, PO_4_-P, NH_4_-N, and TOC. Correlations between these parameters and the multidrug, beta-lactam, phenicol, trimethoprim, MLSB, fluoroquinolone, sulfonamide, rifamycin, tetracycline, and MGEs were stronger than that with rest of the groups (Figure 7B).

### Land use pattern and correlation with antibiotic resistance genes/mobile genetic elements

The dominant land uses in each sampling location in each river are shown in Supplementary Table 5. Cultivation land was the dominant land use in most of the sampling locations and the rivers particularly in TG, CQ, YC, SD, WX, YJP, and DQ. This was followed by either low/medium residential land or Class A/B (CD) River, medium-density residential land, river and cultivation land dominated the rivers followed by either road, river, cultivated land, irrigated pond water or pond water. The medium-density residential land, Class A industrial land and cultivated land dominated the sampling location in Changxing (CX) River and this was followed by the commercial service land, land for green buffer and lake. In Tiaoxi (TX) River, Class A industrial land, medium-density residential land, and cultivated land were the dominant land uses, however, land for green buffer, Class B industrial land, road and river land uses were also represented in high percentages. The Class A industry land, higher education land, medium-density residential land, land for green buffer, cultivated land, irrigated pond water and lake were the dominant land uses in Daqian (DQ) River (Supplementary Table 5).

**FIGURE 8:**
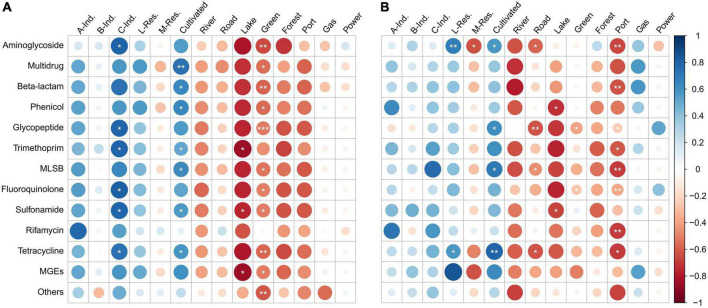
Spearman’s correlation between antibiotic resistance genes (ARGs)/mobile genetic elements (MGEs) absolute abundance and land-use type percentages in **(A)** spring and **(B)** summer, 2019. Larger bubbles indicate higher strengths of correlation than smaller bubbles. Positive correlations are shown in blue, while negative correlations are shown in red. * indicates significance levels of the correlations; *p < 0.05, **p < 0.01, ***p < 0.001. A-Ind, Class A industrial land; B-Ind, Class B industrial land; C-Ind, Class C industrial land; L-Res, Low-density residential land; M-Res, Medium-density residential land; Green, Land for green buffer; Port, Land for port and harbor; Gas, Land for gas stations; Power, Land for Power supply.

The land use values were correlated with the ARGs and MGEs. In spring, all the ARGs and MGEs were positively and strongly correlated with industrial lands use types (Class A and C), low-density residential land and cultivated land (Figure 8A). Correlations between ARGs/MGEs and Class B industrial land was weaker than Class A and C. The ARGs and MGEs had very strong (0.7 < *r* < 1.0) or strong (0.5 < *r* < 0.7) correlations with Class C industrial land. Most of the ARGs/MGEs had very strong (0.7 < *r* < 1.0) or strong (0.5 < *r* < 0.7) correlations with cultivated land. Exceptions were rifamycin and others (0 < *r* < 0.3 weak positive). Negative correlations were observed between most of the ARGs/MGEs and land types including medium-density residential land, river, road, lake, land for green buffer, forest land, land for port and harbor. More than half of the ARGs/MGEs had very strong (−1.0 < *r* < −0.7) or strong (−0.7 < *r* < −0.5) correlations with lake, land for green buffer and forest. Overall, the correlation pattern was stronger and more significant in spring than in summer.

In summer, a similar correlation pattern was observed as in spring. However, the correlations were not as strong as that in spring (Figure 8B). Positive correlations occurred between most of the ARGs/MGEs and industrial lands (Class A, B, and C), low-density residential lands, cultivated lands, and gas station. Very strong (0.7 < *r* < 1.0) or strong (0.5 < *r* < 0.7) positive correlations were observed in Class A industries with phenicol and rifamycin; Class C industries with trimethoprim, MLSB and rifamycin; Low-density residential land with aminoglycoside, tetracycline, and MGEs; cultivated land with aminoglycoside, glycopeptide, MLSB, tetracycline and MGEs. However, statistical significance was only observed for low-density residential land and cultivated lands. Negative correlations were found between most of the ARGs/MGEs and medium-density residential lands, river, road, lake, land for green buffer, forest land, and land for port and harbor. Very strong (−1.0 < *r* < −0.7) or strong (−0.7 < *r* < −0.5) negative correlations were observed in more than half the ARGs/MGEs with river, lake, and land for port and harbor.

## Discussion

Taihu Lake plays important roles as drinking water sources, flood protection, agricultural, fisheries, and many more. Due to various activities in and around the lake, the water pollution and the algal bloom during summer are major concerns. The rivers/canals that are connected to the Taihu Lake contribute to the pollution of the lake (Zheng et al., 2017; Vadde et al., 2018; Vadde et al., 2019a,b; Jiang et al., 2021). The pollution levels of many of the inflow rivers remain unknown, therefore, this study focused on assessing the water quality and the level of ARGs in 10 major inflow rivers of the Taihu Lake.

### High diversity and abundance of antibiotic resistance genes detected in the inflow rivers

The number of ARGs/MGEs detected in each inflow river was ranged from 105 to 185 (average: 153) in spring and 107 to 180 in summer (average: 151), suggesting that occurrence of ARGs/MGEs were varied among locations. Particularly, the number of ARGs were higher in the NW region of the Taihu Lake as compared to SW region. High detection of ARGs conferring resistance to 11 antibiotic classes has proved the level of ARGs in the inflow rivers and the robustness of the HT-qPCR method used for detection and quantification of ARGs. In a previous study, ARGs ranging from 184 to 192 were detected in two drinking water treatment plants which used Qiantang River (Hangzhou, China) water as the drinking source (Xu et al., 2016). Zheng et al. (2017) profiled ARGs in 13 catchment areas in the East Tiaoxi River and they detected a total number of 236 ARGs conferring resistance to all major classes of antibiotics, however, the unique ARGs observed varied among the catchment area ranged from 84 to 142. In another study, qPCR method was used detect and quantify 11 ARGs and 2 MGEs in sampling locations in the Taige Canal and the results showed that ARGs/MGEs were quantified in all the sampling locations in the canal. In the present study, HT-qPCR method was used and 168 and 180 ARGs/MGEs were detected in Taige Canal (TG) in spring and summer, respectively.

Interestingly, a high number of ARGs were also detected in water samples collected from the protected area of Taihu Lake, which served as control for this study (TH-C). This result is in agreement with the observation made by Wang et al. (2014) in which, high numbers of ARGs were detected in the control samples in Baotou, China (detected 81 ARGs in average). In an another study, a total of 117 ARGs, including the genes conferring resistance to aminoglycoside, chloramphenicol, and beta-lactam antibiotics, were detected in pristine area in Antarctic soil with efflux pump and inactivation as the main mechanisms of resistance (Van Goethem et al., 2018). The protected area is a part of Taihu Lake, in which fishing and recreational activities are prohibited, as the water is drawn and treated for drinking. Although various human activities are not allowed in the protected area, the water from other parts of the lake flow into the protected area which cannot be avoided and this explains the reasons for observing high number of ARGs in the protected area. Therefore, the abundance of ARGs detected in the control locations could be either due to the dissemination of ARGs through mixing of water from unprotected area or from the natural environment (Ouyang et al., 2015).

In this study, a number of resistance genes that belong to major antibiotic classes, including those that are critical for medication such as aminoglycoside and beta-lactam, were detected in the water samples. Approximately, 19% of aminoglycoside and 17% beta-lactam resistance genes were detected in the water samples. The values obtained from this study were slightly lower as compared to a previous study in which 19.1–22.8% of beta-lactam resistance genes and 14.3–16.5% of aminoglycoside resistance genes were detected in raw, finished and tap water in Hangzhou, China (Xu et al., 2016). In another study, 23 ARGs conferring resistance to sulfonamides (4 genes), tetracyclines (6), quinolones (4), macrolides (3), beta-lactam (3), and chloramphenicol (3) were investigated in Ba River, China (Jia et al., 2018) and the results showed that 21 out of 23 ARGs were detected in two sampling events indicating the prevalence of the ARGs in the river water. Wu et al. (2022) profiled 198 ARGs and 12 MGEs in water and sediment samples collected from Chaobai River and reported that the ARGs were dominated by beta-lactam resistance genes and it also showed significant seasonal variation in the abundance (Wu et al., 2022). In terms of resistance mechanisms, the most dominant mechanism of ARGs observed in this study was antibiotic inactivation (46%), followed by efflux pump (18%), target alteration (16%), target protection (11%), target replacement (7%), and these results are in consistent with a previous study, in which antibiotic deactivation and efflux pump were found to be the most dominant antibiotic resistance mechanisms in bacteria in drinking water treatment systems in China (Xu et al., 2016). The above resistance mechanisms were previously well described with examples (Blair et al., 2014; Munita and Arias, 2016).

The absolute abundance of ARGs was ranged between 0 and 9.47 Log10 copies/L, which was lower than the ARGs abundance detected in surface water of Wen-Rui Tang River, Wenzhou, China (Zhou et al., 2017). The abundance of ARGs was higher in the summer as compared to spring. An earlier study has observed highest absolute abundance of ARGs in Zhangxi River, China in summer (2.81 × 10^9^ copies/L), followed by autumn, winter, and spring (Zheng et al., 2018) and indicated that water temperature influences the bacterial community dynamics (Kim et al., 2014) and shift in the ARGs profiles. Moreover, the abundance of ARGs was significantly (*p* < 0.05) higher in sampling sites located in the NW region of the Taihu Lake, which was in synergy with a previous study, which showed locations in NW region of the Taihu Lake were most polluted (Qin et al., 2007) and such findings suggest that spread and dissemination of ARGs were significantly influenced by inflow rivers located in the NW region of the of Taihu Lake. Highest ARGs were detected in Shedu River (SD) and Wuxi River (WX), which are surrounded by residential area and villages and also small scale ferry ports are located in these rivers. A previous study has reported highest level of ARGs in water samples collected from town around Zhangxi River, followed by farmland and village, in which human activities were found to influence the abundance of ARGs (Zheng et al., 2018). Additionally, a previous study conducted during 2015 to 2017 reported that no *mcr-1* gene was detected in any of water samples collected from north side of Taihu Lake (Stange et al., 2019). However, the *mcr-1* gene has been detected and quantified in the Haihe River in China and the concentrations were in the range of 3.0 × 10^3^ to 3.8 × 10^5^ copies/L (Yang D. et al., 2017). In the current study, *mcr-1* gene (classified as “others”) was quantified from all inflow rivers, with concentrations ranging from 7.88 × 10^2^ to 3.94 × 10^3^ copies/L and this is a notable finding.

The dissemination of ARGs in water environment highly occurs due to horizontal gene transfer *via* MGEs, including plasmid, integrase, insertional, and transposase (Pruden et al., 2013). In this study, the absolute abundance of MGEs were high in each inflow river, which suggested potential transfer of ARGs among natural aquatic microbial flora. This suggestion was then proven by further analysis in which MGEs were significantly correlated (*p* < 0.05) with most of the absolute abundance of ARGs (Supplementary Figure 4). A previous study by Zheng et al. (2017) reported positive correlation between MGEs and ARGs abundance (*p* < 0.05), specifically to sulfonamide, tetracycline and vancomycin. Wu et al. (2022) found a linear correlation between the abundance ARGs and MGEs in both water and sediments collected from Chaobai River. The significant correlation between MGEs and ARGs indicates the dissemination and proliferation of ARGs may be influenced by MGEs and ARGs conferring resistance to different antibiotic classes can be easily transferred among the bacteria in the water bodies.

### Physico-chemical parameters influence the water pollution and correlates with antibiotic resistance genes

In this study, the physico-chemical analysis of inflow rivers of Taihu Lake was carried out to assess the water quality and its impact on the distribution of ARGs in inflow rivers. The variation in water temperature in both seasons and spatial was significant (*p* < 0.0001), which is obvious due to the sampling months. The pH level in water bodies plays important roles in chemical and biological processes of aquatic organisms (Raveen and Daniel, 2010) and measuring pH level in aquatic systems is important for indicating the water quality and pollution level. The pH of water samples for all inflow rivers in two seasons were within the acceptable limits in both spring and summer. Conductivity is one of the important factors to assess the water quality, which is usually affected by type of soils and presence of inorganic dissolved solids. High value of conductivity in water could be caused by the chloride, nitrate, nitrite, ammonia, and phosphate ions from wastewater discharge or domestic sewage (de Sousa et al., 2014; US EPA, 2017; Vadde et al., 2018; Sekar et al., 2021). In this study, the conductivity values extremely varied in some locations in spring, especially to the sampling locations that are close to residential and businesses. In addition, conductivity values were much lower in summer, which might be due to dilution of water by rainfall event in summer. The rainfall in Suzhou in March, April, and May (Spring 2019) was 50.4, 57.3, and 61.1 mm, respectively, whereas in June, July, and August (Summer 2019) was 127, 129.3, and 247.4 mm, respectively (Suzhou Statistics Bureau, 2020). The protected area of the Taihu Lake (Control, TH-C), ranked the least in the levels of multiple nutrients, which might be due to the protection of this area from human activities as this water is used as source for drinking. High nutrient levels were mainly observed in NW of the Taihu Lake where the ferry transportation activities and discharge of industrial waste, domestic sewage discharge, and animal manure were reported (Zhang et al., 2011). The TN and TP levels exceeded the acceptable limits in all inflow rivers with highest levels in Taige Canal which is considered as one of the major contributors of pollutants to the Taihu Lake. These locations were surrounded by high residential area and industries. Source of elevated TN and TP concentrations might be derived from various sources such as wastewater effluent, runoff from agricultural land and animal manure and industrial discharge (United States Environmental Protection Agency, 2001; Mieszkin et al., 2009). This is consistent with the observation made by Wang et al. (2007), in which high levels of TN and TP was observed in Shedu River and Taige Canal, respectively. NH_4_-N is one of the important sources for ammonification of organic matter (Vaishali and Punita, 2013) and in the current study, NH_4_-N levels were higher in spring than summer, due to high temperature which was more favorable for nitrification process that led to decreased NH_4_-N levels in summer (Vaishali and Punita, 2013). PO_4_-P and Chl *a* levels known to be an indicator for algal blooms, which was proven by significant (*p* < 0.01) correlation between parameters.

Most of the water quality parameters showed stronger and more significant correlations with ARGs/MGEs in spring and the correlation pattern was less obvious in summer. The evolution of ARB and their dispersal are depending on the water environment and this act as ideal medium for bacteria from different origins to mix, exchange and shuffle genes, genetic platforms, and genetic vectors (Baquero et al., 2008). There are considerable evidences that abiotic factors play an important role in the abundance of ARGs in the aquatic environments that are impacted by anthropogenic activities (Jia et al., 2018; Zhang Y. et al., 2021). Similar to the results observed in the present study, significant correlation between the nutrients such as TP, TN, NO_3_-N, NO_2_-N, and NH_4_-N and the ARGs were found in water samples collected from an urban river (Chaobai River, Beijing) in autumn and spring (Wu et al., 2022). The variance partitioning analysis revealed that the environmental parameters such as water temperature, dissolved oxygen, nutrients, metals, and antibiotics contributed to 55–80% and 27–67% of variations of the ARGs and MGEs, respectively (Wu et al., 2022).

### Land use pattern influences the abundance of antibiotic resistance genes/mobile genetic elements

Most of the ARGs/MGEs were positively and strongly correlated with industrial land use (Class A and C), low-density residential land and cultivated land in spring. Similarly, in summer, positive correlations were found between most of the ARGs/MGEs and industrial lands (Class A, B, and C), low-density residential land and cultivated land. As shown in the results, cultivation land was the dominant land use (followed by either low/medium-density residential land or Class A/B industrial land) in seven rivers, in which four located in NW (TG, CQ, YC, and SD) and three in the SW (WX, YJP, and DQ) region of the Taihu Lake. Yang et al. (2022) studied the distribution and abundance of ARGs in human-intensive watershed (including city, river, and lake systems) and found high abundance of ARGs in surface water and sediments with dominance of ARGs conferring resistance to aminoglycoside, beta-lactam and multidrug resistance genes. High diversity of ARGs in spring and summer and high abundance of ARGs in winter were observed and their study concluded that wastewater and human/animal feces contributed to high abundance of ARGs (Yang et al., 2022). The effect of agriculture land-use change on the number and abundance of ARGs in Taihu Lake watershed was studied by Zhang M.-S. et al. (2021) and the results showed that more diverse and higher abundance of ARGs were observed in orchard runoffs as compared to the conventional cropland runoffs particularly when the application of organic manure was higher. In addition, the agricultural land change was reported to aggravate the dissemination of ARGs through the surface run-off (Zhang M.-S. et al., 2021). The number of ARGs detected and the absolute abundance of ARGs were relatively higher in the rivers located in the NW region (TG, CQ, YC, SD, and CD rivers) and this also coincides with high levels of nutrients observed in these rivers. The cultivation, residential and industrial lands around the above sampling rivers might have contributed to high levels of ARGs and nutrients in those rivers. The run-off from those sources should be controlled in order to reduce to the nutrients and the ARGs in the inflow rivers of the Taihu Lake.

## Conclusion

The diversity and the abundance of ARGs in the inflow rivers of Taihu Lake were assessed in this study by using HT-qPCR and the results were correlated with the nutrients and land use patterns. The number and abundance of ARGs and nutrient levels were relatively higher in the rivers located in the NW region of the Taihu Lake as compared to SW region. High abundance of aminoglycoside, beta-lactam, MLSB, multidrug resistance genes were detected. The high abundance and significant correlation between ARGs and MGEs observed in this study shows potential gene transfer among organisms through horizontal gene transfer *via* MGEs. The ARGs detected showed positive correlation with multiple nutrients and the correlation was more prominent in spring as compared to summer. Most ARGs positively correlated with certain land uses such as industrial land, low-density residential land, and cultivated land. The level of nutrients observed in the inflow rivers were above the acceptable limit which indicates poor water quality of the inflow rivers. The input of agriculture run-off, the residential and industrial wastewater should be treated to reduce the pollution in the inflow rivers and eventually the Taihu Lake.

## Data availability statement

The original contributions presented in this study are included in the article/Supplementary material, further inquiries can be directed to the corresponding author.

## Author contributions

RS conceived and designed the experiments. RS, PF, and TY carried out the field sampling. PF performed the experiments with the supervision of RS. JL carried out the land use analysis. SL and BR performed the data analyses. PF, RS, and SL prepared the manuscript. All authors contributed to the revision of the manuscript.
