# Supplementary material for: Diversity and abundance of antibiotic resistance genes and their relationship with nutrients and land use of the inflow rivers of Taihu Lake
Source: Front Microbiol. 2022 Oct 4;13:1009297. doi: 10.3389/fmicb.2022.1009297 (PMC9577174; doi:10.3389/fmicb.2022.1009297)
Supplement: Supplementary file 1 [file Table_1.docx]

Supplementary Material

**Diversity and abundance of antibiotic resistance genes and their relationship with nutrients and land use of the inflow rivers of Taihu Lake**

**Prilli Arista Fernanda^1^, Shuang Liu^1^, Tianma Yuan^1^, Bharathi Ramalingam^2^, Jing Lu^3^ and Raju Sekar^1^***

^1^ *Department of Biological Sciences, Xi’an Jiaotong-Liverpool University, Suzhou, China.*

^2^ *Suzhou Xishan Biotechnology Inc. (VRL Asia), Suzhou, China.*

^3^ *Marie Skłodowska-Curie Actions, SDGine for Healthy People and Cities, Department of Forestry and Environmental Management, Technical University of Madrid (UPM), Madrid, Spain.*

*Correspondence: Raju Sekar: [Sekar.Raju@xjtlu.edu.cn](mailto:Sekar.Raju@xjtlu.edu.cn)

**Table S1.** Summary of the targeted 384 antibiotic resistance genes (ARGs) and mobile genetic elements (MGEs) along with 16S rRNA genes in this study.

| **Gene Group** | **Type** | **Target gene** |
| --- | --- | --- |
| Aminoglycoside resistance | ARGs (*n*=61) | *aac(3)-ib, aac(3)-id_ie,aac(3)-iid_iii_iif_iia_iie, aac(3)-xa, aac(6')I1,aac(6')-Ib, aac(6)-ig, aac(6')-II,* *aac(6)-iic, aac(6)-ij, aac(6)-im, aac(6)-ir, aac(6)-is_iu_ix, aac(6)-iv_ih, aac(6)-iw,* *aac(6')-Iy, aac(6)-iz, aac3ia, aac3-Via, Aac6-Aph2, aacA_aphD, aacA43, aacC2, aadA_99, aadA10, aadA16, aadA17, aadA2-1, aadA2-2, aadA5, aadA6, aadA7, aadA9, aadB, aadD, aadE, acc3-iva, ant4-ib, ant6-ia, ant6-ib, aph(2')-Id, aph(3'')-ia, aph_viii, aph3-ib, aph3-III, aph3via, aph3-viia, aph4-ia, aph4ib, aph6ia, aph6ic, aphA1, aphA3, apmA, ArmA, lsa(C), sat4, spcN, spec_aph, str, strA* |
| β-lactam resistance | ARGs (*n*=54) | *ampC, ampC_blaDHA, beta_ccra, bl1acc, bl3_cpha, bla1, blaACC-1, bla-ACT, blaB-11_13_14, blaCARB, blaCMY, blaCTX-M, blaCTX-M-1_3_15, blaFOXnew, blaGES, blaGOB, blaHERA, blaIMI, blaIMIR, blaIND, bla-L1, blaLEN, blaMIR, blaMOX_blaCMY, blaOCH, blaOXA10, blaOXY-1, blaOXY-2, blaPAO_PDC, blaPER, blaPSE, blaROB, blaSFO, blaSHV-11, bla-SME, blaTEM, blaTLA, blaVEB, blaVIM, blaZ, cefa_ampc, cepA, cfiA, cfxA, cphA, KPC, imp-marko, mecA, NDM new, nonmobile blaBEL, nonmobile_blaADC, pbp, Pbp5, penA* |
| Phenicol resistance | ARGs (*n=15*) | *Cat, cat(pC221), catA1, catA2, catA3, catB2, catB3, catB8, catB9, catP, catQ, ceoA, cmlA5, cmlV, optrA* |
| Glycopeptide resistance | ARGs (*n=24*) | *vanA, vanB, vanC, vanC2_vanC3, vanD, vanG, vanHB, vanHD, vanRA, vanRB, vanRC, vanRC4, vanRD, vanSA, vanSB, vanSC, vanTC, vanTE, vanTG, vanWB, vanXA*  *vanXB, vanYB, vanYD* |
| Trimethoprim resistance | ARGs (*n*=19) | *dfrA1, dfrA10, dfrA12, dfra14, dfrA15, dfra17, dfrA18, dfra21, dfrA22, dfrA25, dfrA27, dfra5, dfra7, dfrA8, dfrAB4, dfrBmulti, dfrC, dfrG, dfrK* |
| Macrolide-, lincosamide- and streptogramins B (MLSB) resistance | ARGs (*n*=44) | *carB, cfr, ere(A), ere(B), erm(34), erm(35), erm(36), erm(42), erm(A), erm(B), erm(D), erm(E), erm(F), erm(G), erm(O), erm(Q), erm(S), ermA_ermTR, ermK, ermT, ermX, ermY, lmrA, lnu(F), lnuA, lnuB, lnuC, mef(B), mefA, mphA, mphB, msr(A), msr(C), msr(D), msr(E), oleC, pica, pikR2, vat(A), vat(E), vatB, vga(A)LC, vgaA, vgaB* |
| Fluoroquinolone resistance | ARGs (*n=10*) | *oqxA, qnrA, QnrB4, qnrB46_47_48, qnrB-bob_resign qnrD, QnrS1_S3_S5, qnrS2, QnrVC1_VC3_VC6 QnrVC4_VC5_VC7* |
| Sulfonamide resistance | ARGs (*n*=6) | *folA, strB, sul1 NEW, sul2, sulA_folP, sulIII* |
| Rifamycin resistance | ARGs (*n*=2) | *Arr2, ARR-3* |
| Tetracycline resistance | ARGs (*n*=27) | *tet(32), tet(36), tet(38), tet39, tet40, tet44, tetA, tetB, tetbP, tetC, tetD, tetE, tetG_F, tetH, tetJ, tetK, tetL, tetM tetO, tetPA, tetPB, tetQ, tetR, tetS, tetT, tetW, tetX* |
| Multidrug resistance (MDR) | ARGs (*n*=49) | *acrA, acrB, acrF, acrR, adeA, adeI, arsA, bexA_norM, cadC, cefa_qacelta, cmlA1, cmr, cmx(A), copA, czcA, emrB_qacA, emrD, fexA, floR, marR, mdtE_yhiU, mdtA, mdtg, mdth, mepA, merA-marko, mexA, mexB, mexE, mtrD, mtrE, multidrug resistance , nimE, norA, oprD, pbrT, pcoA, qacA_B, qacF_H, qacH_351, qepA_1_2, silE, sugE, tcrB, terW, tetU, tolC, ttgA, ttgB* |
| Mobile genetic element (MGE) | MGEs (*n=24*) | *Cro, EAE_05855, IncHI2-smr0018, IncI1_repI1, IncN_korA, IS1247, IS15DI, IS200-1, IS200-2, IS21-ISAs29, IS256, IS26, IS3, IS5_IS1182, IS6_257, IS6100, IS630, IS91, ISCR1, ISEcp1, lncF_FIC, mobA, Tn3, TN5403* |
| Integrase | MGEs (*n*=3) | *int1, intl2, intl3* |
| Plasmid/plasmid-inc/ plasmid-rep | MGEs (*n*=11) | *tra-A, traN, trb-C, IncN_oriT, IncN_rep, IncP_oriT, IncQ_oriT, IncW_trwAB, pAKD1-IncP-1, PAMBL-1-F_377old, pBS228-IncP-1* |
| Insertion sequence | MGEs (*n*=9) | *IS1111, IS1133, ISAba3-Acineto, ISEfm1-Entero, ISPps1-pseud, ISSm2-Xanthob, orf37-IS26, orf39-IS26, TN5* |
| Transposase | MGEs (*n*=10) | *IS613, tnpA-1, tnpA-2, tnpA-3, tnpA-4, tnpA-5, tnpA-6, tnpA-7, Tp614, trfa* |
| Taxonomic | Resistance genes and specific detection (*n*=6) | *ecfX-P. aeruginosa, Firmicutes, gltA-K. pneumonia, mecA-Staphylococci, ompA-A. baumannii, Bacteroidetes* |
| Others | Resistance genes of fosfomycin, colistin, bacitracin, streptothricin, trimethoprim and total bacteria (*n*=10) | *fosB, fosX, fabK, mcr-1, mcr-2, nisB, pmrA, bacA, sat4, folA and 16S rRNA* |

MGEs: mobile genetic elements; ARGs: antibiotic resistance genes

**Table S2.** The ARGs conferring resistance to different antibiotic classes, multidrug and MGEs detected in the inflow rivers and control locations in Spring 2019.

|  | Taige Canal  (TG) | Caoqiao River  (CQ) | Yincun River  (YC) | Shedu River  (SD) | Chendong River  (CD) | Wuxi River  (WX) | Changxing River  (CX) | Yangjiapu River  (YJP) | Tiaoxi River  (TX) | Daqian River  (DQ) | Taihu Lake - Control  TH(C) |
| --- | --- | --- | --- | --- | --- | --- | --- | --- | --- | --- | --- |
| Aminoglycoside | 35 | 38 | 36 | 39 | 31 | 27 | 32 | 29 | 27 | 24 | 20 |
| Beta-lactam | 13 | 18 | 19 | 12 | 14 | 13 | 19 | 13 | 9 | 9 | 7 |
| Phenicol | 7 | 9 | 8 | 8 | 5 | 5 | 5 | 6 | 4 | 3 | 3 |
| Glycopeptide | 8 | 8 | 7 | 8 | 7 | 5 | 5 | 8 | 5 | 4 | 4 |
| Trimethoprim | 7 | 8 | 9 | 8 | 5 | 6 | 7 | 6 | 5 | 3 | 4 |
| MLSB | 21 | 21 | 20 | 20 | 17 | 16 | 17 | 16 | 14 | 14 | 11 |
| Fluoroquinolone | 5 | 6 | 3 | 3 | 4 | 3 | 5 | 4 | 2 | 2 | 2 |
| Sulfonamide | 1 | 1 | 1 | 1 | 1 | 1 | 1 | 1 | 1 | 1 | 1 |
| Rifamycin | 2 | 2 | 2 | 2 | 2 | 1 | 2 | 1 | 2 | 0 | 2 |
| Tetracycline | 10 | 10 | 12 | 9 | 10 | 6 | 10 | 6 | 5 | 5 | 4 |
| Multidrug | 22 | 28 | 28 | 27 | 24 | 27 | 25 | 25 | 21 | 20 | 19 |
| MGEs | 31 | 30 | 33 | 31 | 24 | 23 | 32 | 21 | 18 | 16 | 14 |
| Others | 6 | 6 | 6 | 5 | 5 | 6 | 6 | 6 | 4 | 4 | 5 |
| Total | **168** | **185** | **184** | **173** | **149** | **139** | **166** | **142** | **117** | **105** | **93** |

**Table S3.** The ARGs conferring resistance to different antibiotics classes, multidrug and MGEs detected in the inflow rivers and control locations in Summer 2019.

|  | Taige Canal  (TG) | Caoqiao River  (CQ) | Yincun River  (YC) | Shedu River  (SD) | Chendong River  (CD) | Wuxi River  (WX) | Changxing River  (CX) | Yangjiapu River  (YJP) | Tiaoxi River  (TX) | Daqian River  (DQ) | Taihu Lake - Control  TH(C) |
| --- | --- | --- | --- | --- | --- | --- | --- | --- | --- | --- | --- |
| Aminoglycoside | 39 | 29 | 29 | 27 | 30 | 27 | 28 | 28 | 21 | 25 | 20 |
| Beta-lactam | 19 | 15 | 14 | 16 | 18 | 19 | 17 | 14 | 7 | 16 | 9 |
| Phenicol | 7 | 7 | 5 | 6 | 8 | 5 | 7 | 5 | 3 | 6 | 2 |
| Glycopeptide | 5 | 5 | 6 | 5 | 6 | 8 | 7 | 6 | 4 | 6 | 5 |
| Trimethoprim | 9 | 7 | 6 | 7 | 6 | 7 | 8 | 6 | 6 | 9 | 4 |
| MLSB | 21 | 19 | 17 | 20 | 20 | 19 | 16 | 16 | 16 | 15 | 14 |
| Fluoroquinolone | 5 | 6 | 5 | 4 | 4 | 4 | 3 | 4 | 2 | 3 | 2 |
| Sulfonamide | 1 | 2 | 1 | 2 | 1 | 1 | 1 | 1 | 1 | 1 | 1 |
| Rifamycin | 2 | 2 | 2 | 2 | 2 | 2 | 2 | 2 | 2 | 2 | 1 |
| Tetracycline | 12 | 6 | 6 | 10 | 12 | 8 | 9 | 6 | 4 | 6 | 7 |
| Multidrug | 26 | 22 | 23 | 24 | 26 | 26 | 27 | 27 | 20 | 21 | 18 |
| MGEs | 29 | 28 | 23 | 29 | 29 | 22 | 28 | 24 | 15 | 21 | 20 |
| Others | 5 | 6 | 5 | 6 | 6 | 6 | 6 | 7 | 6 | 6 | 6 |
| Total | **180** | **154** | **142** | **158** | **168** | **154** | **159** | **146** | **107** | **137** | **107** |

Table S4. Statistical analyses for absolute and relative abundance of ARGs conferring resistance to different antibiotic classes, multidrug and MGEs in the inflow rivers.

| Antibiotic classes | Absolute abundance | | Relative abundance | |
| --- | --- | --- | --- | --- |
|  | Seasonal | Spatial | Seasonal | Spatial |
| Aminoglycoside | 0.5633 | 0.2122 | 0.6222 | 0.0098** |
| Beta-lactam | 0.6508 | 0.0934 | 0.2252 | 0.039* |
| Phenicol | 0.7058 | 0.0055** | 0.3449 | 0.0001*** |
| Glycopeptide | 0.9325 | 0.0437* | 0.3589 | 0.0046** |
| Trimethoprim | 0.3236 | 0.0285* | 0.5086 | 0.0022 |
| MLSB | 0.2774 | 0.0922 | 0.1783 | 0.0725 |
| Fluoroquinolone | 0.4410 | 0.0091** | 0.0555 | 0.003** |
| Sulfonamide | 0.0789 | 0.0470* | 0.0325* | 0.0568 |
| Rifamycin | 0.0037** | 0.0052** | 0.0303* | 0.0108* |
| Tetracycline | 0.9440 | 0.1845 | 0.3638 | 0.0161* |
| Multidrug | 0.2752 | 0.0032** | 0.0545 | 0.0073** |
| MGEs | 0.7915 | 0.0755 | 0.33767 | 0.0042 ** |
| Others | 0.0071 ** | 0.3492 | 0.5860 | 0.2250 |

*Statistically significant difference at p < 0.05; ** Statistically significant difference at p < 0.01; *** Statistically significant difference at p < 0.001; **** Statistically significant difference at p < 0.0001.

Table S5. Dominant land uses in each sampling location and river along with coordinates of the sampling locations.

| **River** | **Sampling Location** | **Description of Location** | **Coordinates** | |
| --- | --- | --- | --- | --- |
|  |  |  | **Latitude** | **Longitude** |
| A. Taige Canal (TG) | A1 | Cultivated land (66.01%) followed by Low Density Residential land (9.3%), Road (6.76%) and Land for green buffer (5.8%). | N31°31′55.28″ | E120°0′30.68″ |
|  | A2 | Cultivated land (34.95%) followed by Class B industrial land (20.45%), Low-density residential land (11.78%), Class A industrial land (7.05%) and River (5.05%). | N31°31′9.31″ | E120°0′59.02″ |
|  | A3 | Cultivated land (54.46%) followed by Low-density residential land (7.92%), Class A industrial land (6.88%), Commercial service land (5.92%), Roads (5.41%) and River (5.31%). | N31°29′41.26″ | E120°01′32.96″ |
| B. Caoqiao River (CQ) | B1 | Cultivated land (19.04%) followed by Class B industrial land (12.95%), Low Density Residential land (12.31%), Commercial service land (11.73%), Class C industrial land (9.6%), Road (8.87%), Medium-density residential land (7.41%) and Class A industrial land (6.29%). | N31°31′6.61″ | E119°58′17.86″ |
|  | B2 | Cultivated land (41.91%) followed by Class A industrial land (11.4%), Class B industrial land (11.2%), Low-density residential land (8.24%), Commercial service land (5.38%) and Road (5.01%). | N31°29′41.57″ | E120°0′58.80″ |
|  | B3 | Cultivated land (43.07%) followed by Class A industrial land (10.19%), Class B industrial land (8.42%), Low-density residential land (7.5%), Road (6.43%), Commercial service land (6.13%) and River (5.67%). | N31°29′30.28″ | E120°01′35.10″ |
| C. Yincun River (YC) | C1 | Medium-density residential land (26.1%) followed by Cultivated land (23.3%), Class A industrial land (14.79%), River (8.79%) and Road (6.86%). | N31°28′28.06″ | E119°56′20.37″ |
|  | C2 | Cultivated land (49.62%) followed by Medium-density residential land (10.04%), Public green park (7.69%), Road (7.53%), Class C industrial land (7.08%) and River (5.15%). | N31°27′39.97″ | E119°58′59.31″ |
|  | C3 | Cultivated land (71.03%) followed by Low-density residential land (8.21%) and River (7.6%). | N31°27′10.64″ | E120°0′29.86″ |
| D. Shedu River (SD) | D1 | Cultivated land (58.32%) followed by Class B industrial land (9.82%), Low-density residential land (9.69%) and River (8.63%). | N31°22′45.80″ | E119°53′51.10″ |
|  | D2 | Cultivated land (43.5%) followed by Class A industrial land (18.48%), River (7.88%), Low-density residential land (5.83%), Logistic and Warehousing land (5.69%), Land for green buffer (5.50%) and Irrigated pond water (5.07%). | N31°22′20.65″ | E119°54′43.46″ |
|  | D3 | Cultivated land (66.83%) followed by Low-density residential land (19.84%). | N31°21′19.88″ | E119°56′43.10″ |
| E. Chendong River (CD) | E1 | Medium-density residential land (47.32%) followed by Road (12.89%), River (12.50%) and Public green park (7.73%). | N31°21′14.10″ | E119°50′10.98″ |
|  | E2 | River (50.95%) followed by Cultivated land (28.2%), Irrigated pond water (10.4%) and Land for green buffer (5.08%). | N31°20′40.81″ | E119°52′15.25″ |
|  | E3 | Cultivated land (36.46%) followed by Pond water (12.21%), Medium-density residential land (12.01%), Low-density residential land (6.37%), River (6.18%), Road (6%), Land for green buffer (5.21%) and Class A industrial land (5.08%). | N31°18′52.95″ | E119°55′25.29″ |
| F. Wuxi River (WX) | F1 | Cultivated land (43.33%) followed by Class A industrial land (11.84%), Medium-density residential land (11.05%), Low-density residential land (9.05%) and River (7.07%). | N31°02′42.80″ | E119°55′26.96″ |
|  | F2 | Cultivated land (60.54%) followed by River (11.54%), Low-density residential land (10.53%) and Forest land (7.33%). | N31°03′12.56″ | E119°57′4.85″ |
|  | F3 | Lake (36.8%) followed by Cultivated land (26.4%), Forest land (11.13%) and Low-density residential land (8.19%). | N31°03′24.29″ | E119°58′48.06″ |
| G. Changxing River (CX) | G1 | Medium-density residential land (34.41%) followed by Commercial service land (25.81%), Road (12.54%), Low-density residential land (7.04%) and River (5.41%). | N31°0′6.29″ | E119°54′17.30″ |
|  | G2 | Class A industrial land (28.05%) followed by Cultivated land (25.76%), Land for green buffer (10%), Road (9.66%) and River (8.12%). | N31°0′33.67″ | E119°56′22.26″ |
|  | G3 | Cultivated land (28.06%) followed by Lake (27.18%), Low-density residential land (9.93%), Medium-density residential land (8.41%), Road (8.29%) and River (7.38%). | N31°02′5.60″ | E119°59′16.48″ |
| H. Yangjiapu River (YJP) | H1 | Cultivated land (27.49%) followed by Class B industrial land (21.04%), Low-density residential land (14.97%), River (9.01%) and Road (5.93%). | N30°58′7.62″ | E119°57′55.09″ |
|  | H2 | Cultivated land (63.88%) followed by River (13.16%) and Low-density residential land (9.96%). | N30°59′33.10″ | E119°59′53.55″ |
|  | H3 | Lake (40.48%) followed by Cultivated land (26.22%), Forest land (9.05%), Low-density residential land (6.32%), Pond water (6.07%), Road (5.54%) and River (5.25%). | N31°01′9.38″ | E120°0′40.27″ |
| I. Tiaoxi River (TX) | I1 | Class A industrial land (15.92%) followed by Land for green buffer (15.49%), Road (15.32%), Class B industrial land (11.46%), Land for port and harbor (9.82%), River (7.65%), Reserved land (7.62%) and Cultivated land (5.61%). | N30°53′23.14″ | E120°02′18.39″ |
|  | I2 | Medium-density residential land (31.27%) followed by River (17.2%), Road (9.07%), Public green park (7.87%), Land for green buffer (7.63%), Cultivated land (7.18%) and Reserved land (5%). | N30°53′51.23″ | E120°05′54.11″ |
|  | I3 | Cultivated land (30.69%) followed by Medium-density residential land (18.62%), River (16.13%), Road (9.94%), Lake (7.36%) and Low-density residential land (5.27%). | N30°56′25.23″ | E120°07′38.86″ |
| J. Daqian River (DQ) | J1 | Class A industrial land (13.74%) followed by Higher education land (12.49%), Medium-density residential land (11.69%), Land for green buffer (10.34%), Class B industrial land (8.3%), River (8.2%), Commercial service land (8.05%), Secondary vocational education land (7.88%) and Road (7.38%). | N30°52′43.51″ | E120°08′12.66″ |
|  | J2 | Cultivated land (36.21%) followed by Irrigated pond water (26.14%), Low-density residential land (10.47%), Land for green buffer (9.83%), Road (8.02%) and River (6.66%). | N30°55′5.24″ | E120°09′39.98″ |
|  | J3 | Lake (43.5%) followed by Cultivated land (34.2%) and Irrigated pond water (8.21%). | N30°55′52.18″ | E120°11′34.52″ |


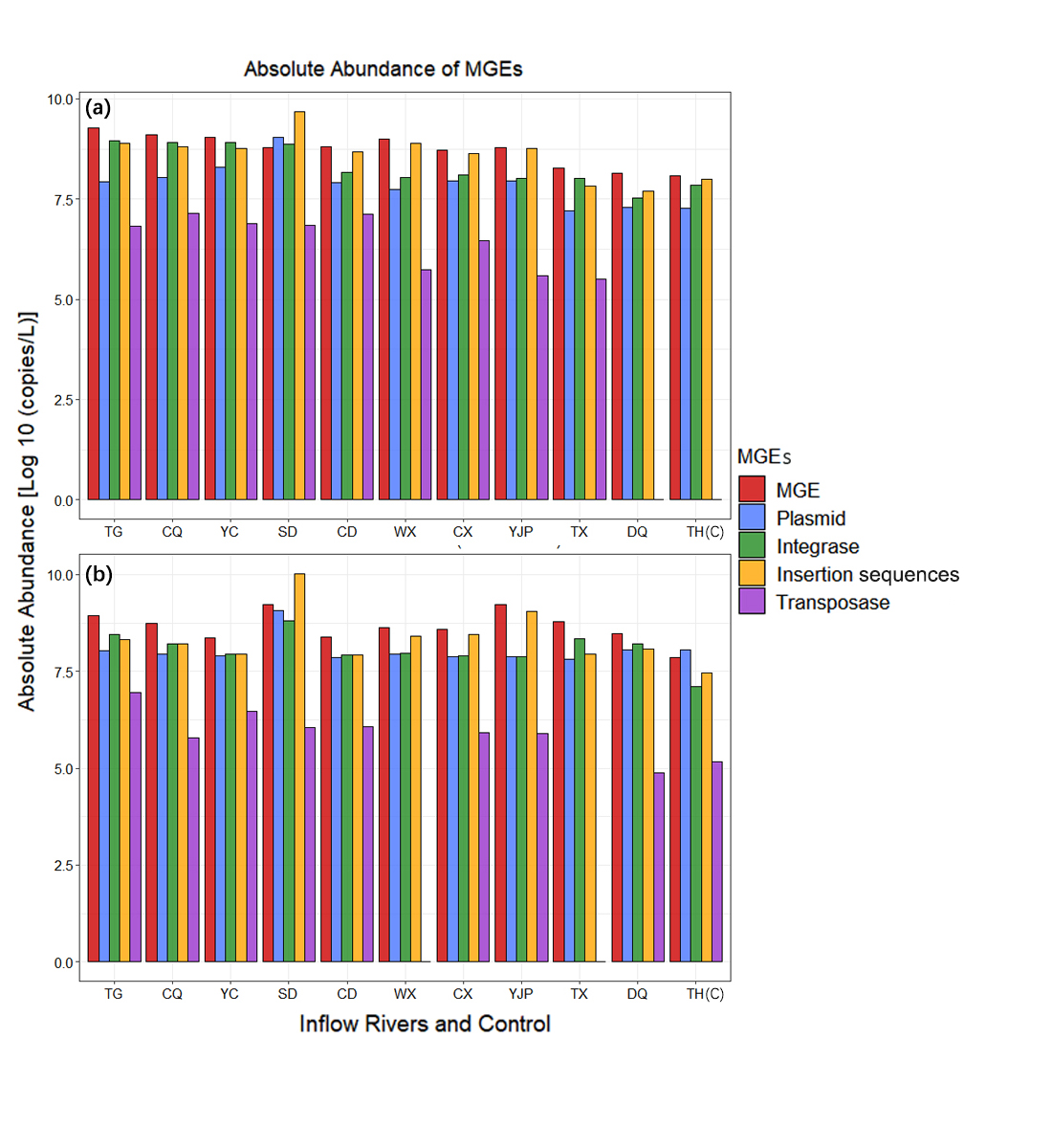


**Figure S1**. Absolute abundance of mobile genetic elements (MGEs) in the inflow rivers and control location of Taihu Lake in (a) spring and (b) summer.

**Figure 5**


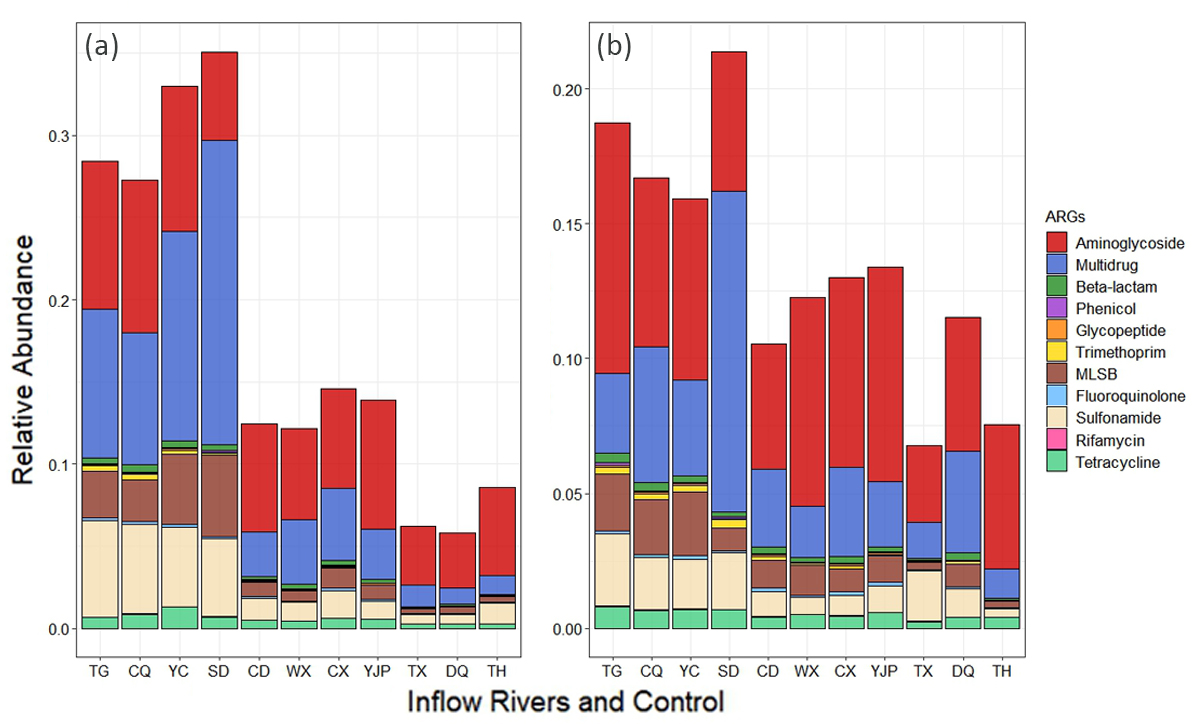


**Figure S2**. Relative abundance of ARGs (grouped based on the antibiotic class to which they confer resistance) in inflow rivers and control location during (a) spring and (b) summer.


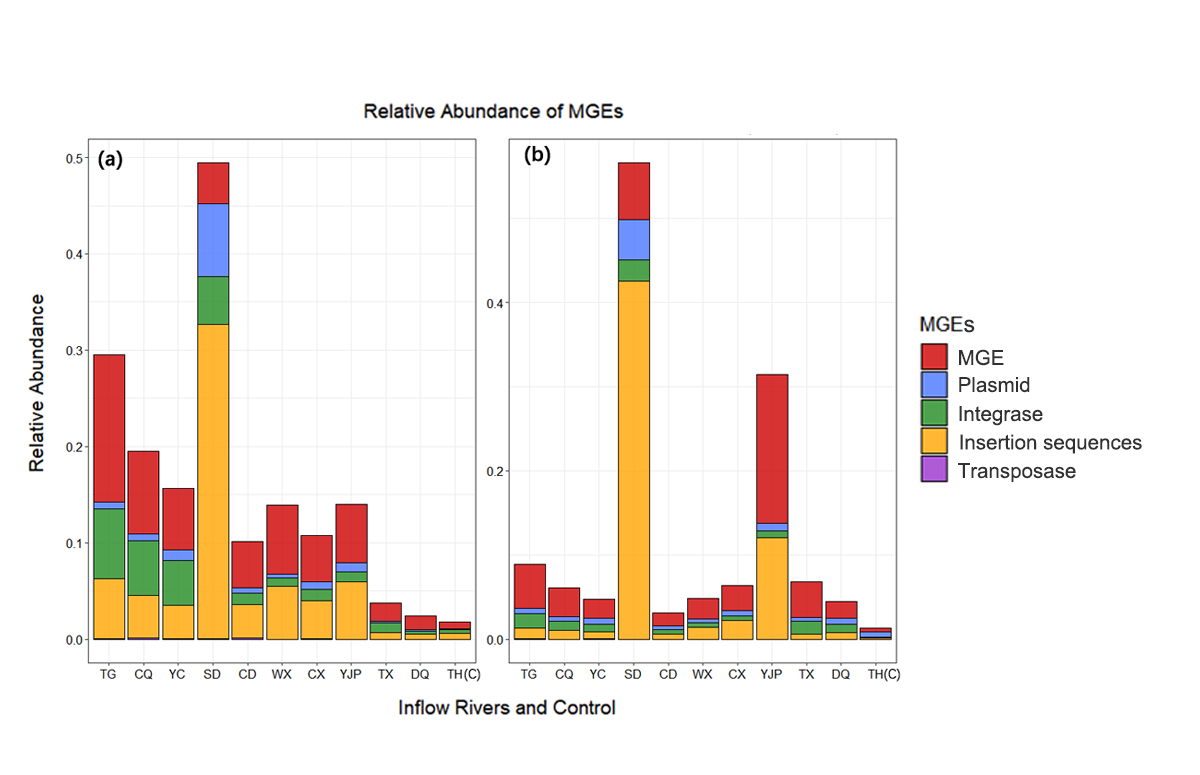


**Figure S3.** Relative abundance of MGEs in the inflow rivers and control location of Taihu Lake in (a) spring and (b) summer.


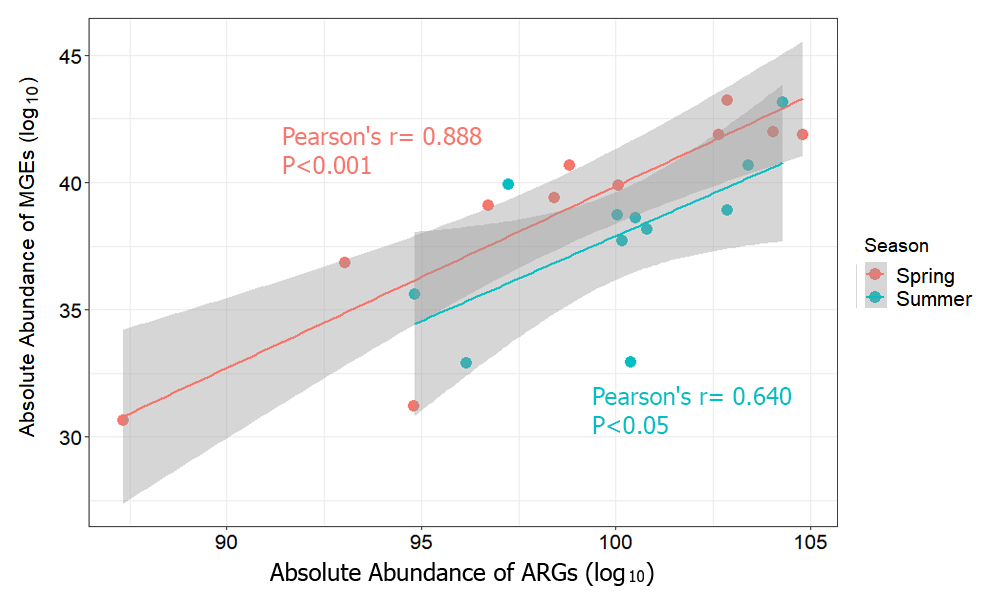


**Figure S4.** Pearson correlation of absolute abundance of ARGs with absolute abundance of MGEs.
