# Supplementary material for: Diversity and abundance of antibiotic resistance genes and their relationship with nutrients and land use of the inflow rivers of Taihu Lake
Source: Front Microbiol. 2022 Oct 4;13:1009297. doi: 10.3389/fmicb.2022.1009297 (PMC9577174; doi:10.3389/fmicb.2022.1009297)
Supplement: Supplementary file 2 [file Presentation_1.pdf]

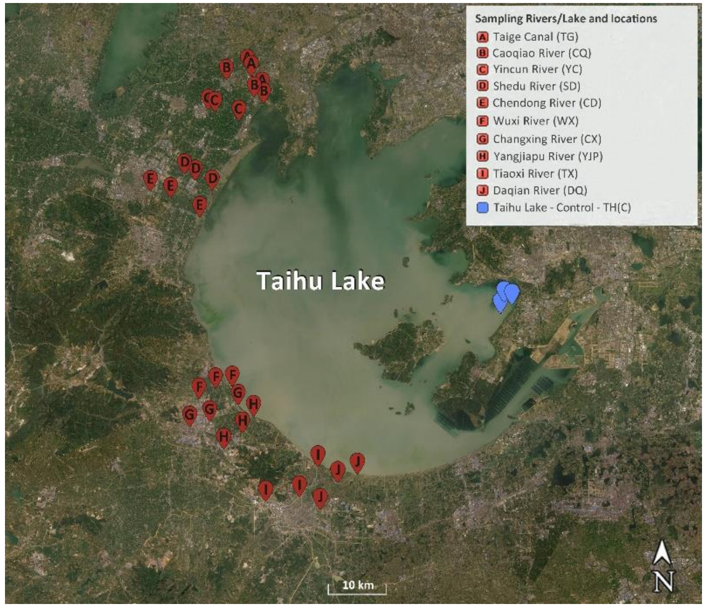

Sampling locations

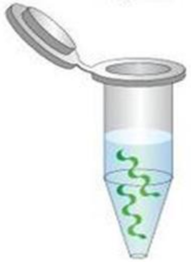

DNA extraction

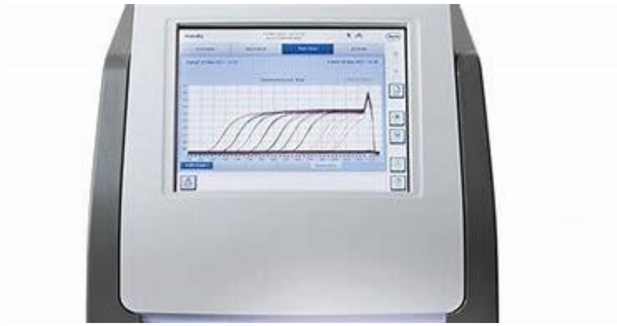

HT-qPCR analysis of ARGs

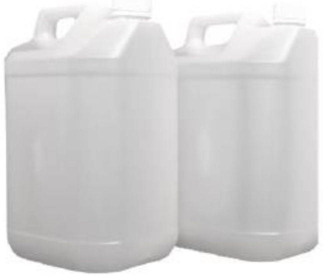

Water sampling

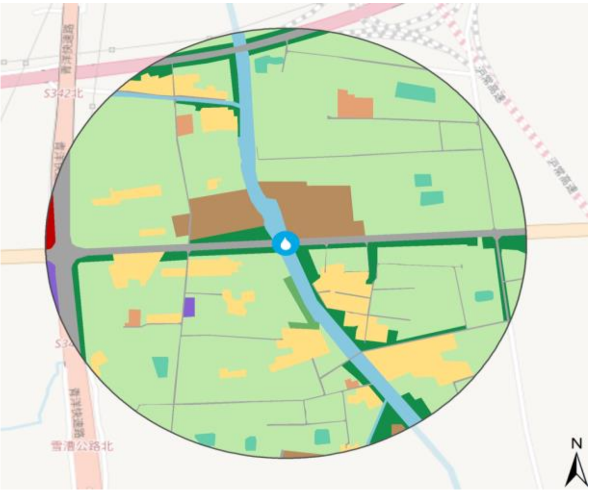

Land use and Water quality analyses

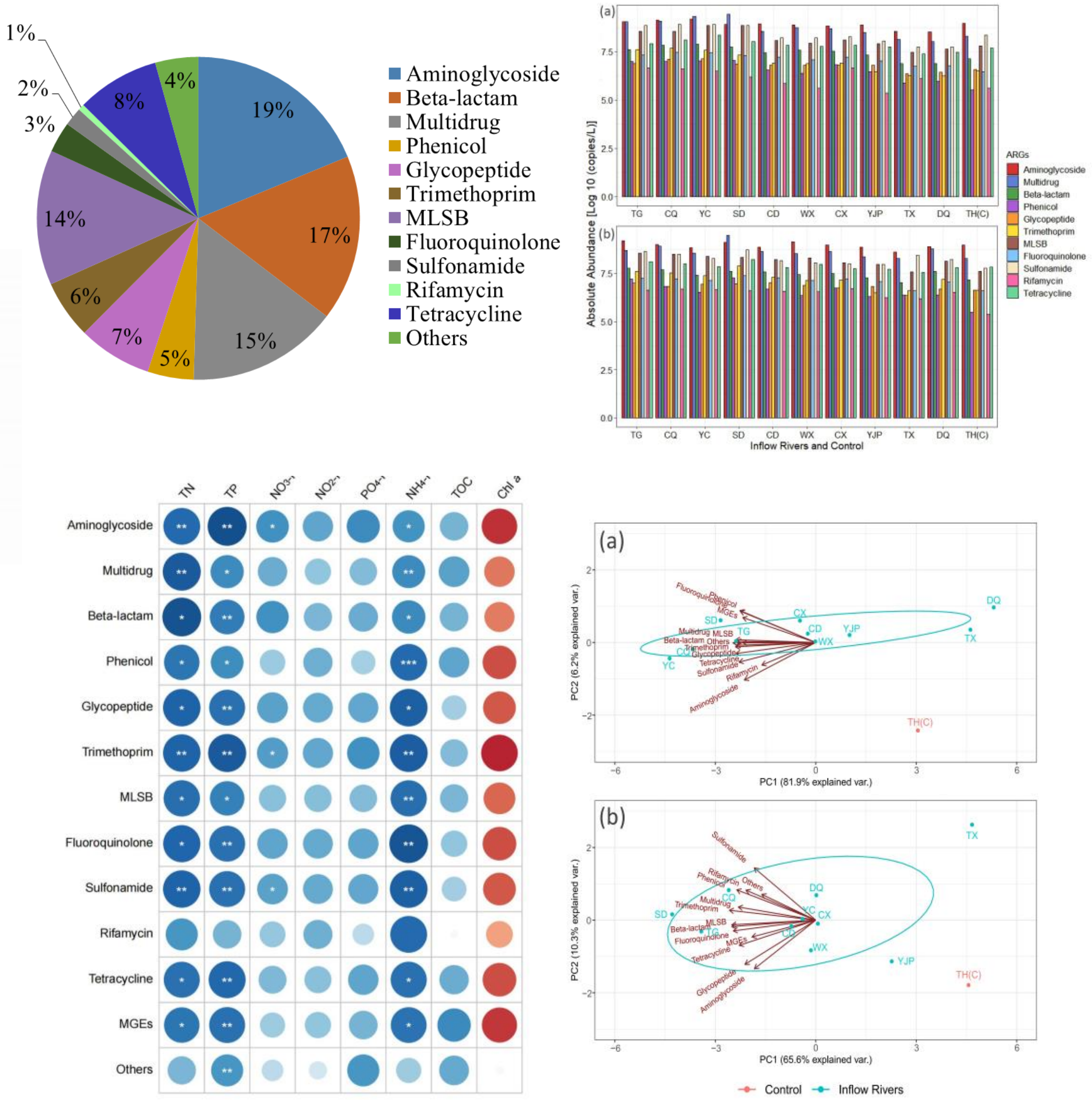

ARGs and their relationship with land use and nutrients
